# Using Sensory Properties of Food to Trigger Swallowing: A Review

**DOI:** 10.1080/10408398.2011.649810

**Published:** 2014-08-08

**Authors:** C. Loret

**Affiliations:** ^a^ Nestlé Research Center, P.O. Box 44, Vers-Chez-Les-Blancs, CH-1000 Lausanne 26, Switzerland

**Keywords:** Food diet, food perception, oral cavity, dysphagia

## Abstract

The effect of food consistency on swallowing function has been widely studied, and it is well recognized that by delaying the flow of the food bolus, thickened liquids can help in the management of swallowing dysfunction. However, fewer studies have been carried out on the impact of food sensory properties and related liking on swallowing function. This paper reviews the role of taste, olfaction, and trigeminal perceptions on swallowing function and highlights the need for a deeper investigation of this aspect of patient diet modification.

## 1. INTRODUCTION

Swallowing is a mandatory step for foods and liquids to be ingested. Consequently, difficulty in swallowing, clinically termed “dysphagia,” can lead to dehydration and malnutrition. A poor muscle coordination during swallowing, a delayed swallow response and/or residue of bolus material can also lead to aspiration, which can result in pneumonia, above all when malnutrition is present. To reduce these complications, thickened liquids are recommended as a dietary treatment (American Dietetic Association, 2002). These are prescribed based on their physical properties (primarily the viscosity/consistency), which are believed to module the flow of liquids as they enter the pharynx and hence, at least partially, compensate for delayed swallow response.

**Table 1:** Reported effects of food sensory properties on swallowing parameters

							Capsaicin/		Odorant+
	Sour	Salt	Sweet	Bitter	Umami	Carbonation	menthol/pepper	*T* (°C)	Taste
Reduced oral and pharyngeal transit time	Yes^1^	Yes^2^	Yes^2^	No^2^	NS	Yes^1^	Yes^3^	Yes^1,2,4^	Yes^2^
Reduced aspiration and penetration	Yes^1^	NS	NS	NS	NS	Yes^1^	NS	NS	NS
Increased spontaneous dry swallows	NS	NS	NS	NS	NS	NS	NS	NS	Yes^2^
Stronger and shorter submental muscle contraction	Yes^2^	Yes^2^	Yes	Longer muscle contraction	NS	Yes^2^	Yes^3^	NS	NS
High lingual pressure	Yes^1^	Yes^1^	Yes^1^	No^1^	NS	NS	NS	NS	NS
Effect on duration of swallowing apnea	No^2^	No^2^	No^2^	No^2^	NS	NS	NS	NS	NS

^1^Neurogenic population.^2^Healthy population.^3^Institutionalized elderly patients.^4^Stroke patients.NS = not studied.

However, most practitioners perceive that their patients dislike thickened liquids (Garcia et al., 2005). Patients who have a strong dislike of thickened liquids may, as a result, avoid drinking swallow-safe liquids. On the basis of the retrospective cohort study of 140 patients, Low et al. (2001) showed that noncompliance with the recommended dietary modification for dysphagia is associated with adverse outcomes such as a high mortality rate and aspiration pneumonia. One way to improve patient compliance and thus decrease associated risks is therefore to improve the sensory properties of thickened liquids.

Few articles have been published on the sensory characterization of commercial thickeners used for dysphagia diets (Pelletier, 1997; Lotong et al., 2003; Matta et al., 2006). Pelletier (1997) evaluated the performance of five commercial thickeners (Thick It, Thicken Right, Thick and Easy, ThickenUp, and QuikThick). Her findings suggest that none of the commercial thickeners tested produced a desired consistency and the products were not perceived to be tasty. Matta et al. (2006) also reported that with thickeners, the main flavors of the underlying base beverages (milk, apple juice, and orange juice) were suppressed (certainly due to the sensory interaction texture/flavor or chemical interaction biopolymers/flavor) and imparted slight off-flavors (bitter, sour, metallic, or astringent). Starch-based thickeners (Thick and Easy, Thicken Up) imparted a starchy flavor and a grainy texture, whereas gum-based thickeners (Thick and Clear, Simply Thick) gave an undesirable added slickness to the beverages. The authors conclude that additional development of thickening agents seems necessary if we wish to improve sensory properties.

As of today, no conclusive results have been published that link food liking and swallowing performance. Pelletier and Dhanaraj (2006) did not find any significant effect of taste palatability on lingual swallowing pressure. Miyaoka et al. (2006) investigated the effect of thermal and gustatory food properties on perceived ease of swallowing, and on swallowing motor muscle parameters using submental surface electromyography (sEMG). They could only relate a perceived ease of swallowing to swallowing-related muscles (i.e., suprahyoid muscle) for thickened water consumed at 50°C. Despite a difference in perceived ease of swallowing between the taste solutions tested (sweet, salty, sour, bitter, and umami), no change in swallowing motor parameters was observed. Nevertheless, the authors argue that these results do not mean that there is no effect, because the physiological measurements used are relatively insensitive. Recent results from Miura et al. (2009) also suggest that liking does not influence the swallowing function (i.e., the power frequency of swallowing sEMG). Mistry et al. (2006) hypothesized that changes in taste pleasantness, assuming that sweetness is a pleasant taste and bitterness is an unpleasant taste, would have modulatory effects on swallowing cortical excitability measured by transcranial magnetic stimulation (TMS) mapping before and after solution infusion. Their results did not confirm their hypothesis. However, despite the lack of clinical evidence, it seems likely that specific sensory properties could have a subtle, direct impact on swallowing function beyond hedonic aspects.

Human functional brain imaging studies indicate that the transduction of taste sensations is reliant on multiple brain regions, specifically the orbitofrontal cortex, insula, and amygdale areas that are also activated in swallowing (Teismann et al., 2007; Lowell et al., 2008; Babaei et al., 2010). A recent review (Steele and Miller, 2010) summarized the sensory pathways and mechanisms that are believed to control the different phases of swallowing (oral, pharyngeal, esophageal phases) reinforcing the importance of sensory inputs on swallowing. The present review is focused on the influence of tastants in food matrices on swallowing physiological parameters. We also include the few papers dealing with other food sensory modalities, i.e., olfaction, trigeminal, and texture.

## 2. FOOD SENSORY PROPERTIES WITH A POTENTIAL ROLE IN TRIGGERING THE SWALLOW

To investigate the effect of food sensory properties on swallowing function, we focused our literature review on voluntary human swallowing in the oral cavity. The main physiological effects of food sensory stimuli on swallowing are reported in Table 1.

### 2.1. Sour Perception

Studies have shown that a sour bolus improves swallowing response in neurogenic dysphagic patients (Logemann et al., 1995; Pelletier and Lawless, 2003). The hypothesis given for the impact of taste on swallowing function is that “taste is an important oral sensory stimulus […] that […] might […] increase the pre-swallow sensory input to the cortex and brain stem, thus lowering the swallow threshold.” (Logemann et al., 1995). Lowering the swallowing threshold, i.e., the level of stimulation at which a swallow will be triggered, could decrease the residence time in mouth and speed up the pharyngeal swallow. Logemann et al. (1995) carried out a videofluoroscopic swallow study (VFSS) using either 50% water/50% barium sulfate solution or 50% real lemon juice/50% barium sulfate solution. The sour bolus was shown to improve the onset of the oral swallow. The patients exhibited a number of other improvements in the swallow as a result of the sour bolus, including both reduced oral and pharyngeal transit time, and improved oropharyngeal swallow efficiency (i.e., the ratio between the percentage of food bolus entering into the esophagus and the total oral and pharyngeal transit time). Some patients also exhibited reduced aspiration as a result of the sour bolus. However, in this study, three sensory stimuli were involved and their effect could not be differentiated: sour taste, lemon odorant, and contrast agent flavor. Indeed, by itself the contrast agent has a significant taste, which could alter the way the food tastes. Ekberg et al. (2009) evaluated the ease of swallowing for four mango puree samples of different viscosities (ranging from 1 to 2 Pa·s) with or without added barium sulfate medium and found that samples with high concentration of barium sulfate were perceived to be thicker and more particulate, which probably explains why these samples were perceived as less easy to swallow. On the basis of these observations, the author would not recommend carrying out a VFSS study (where contrast agents are needed) to investigate the effect of sensory properties on swallowing function.

To confirm the triggering effect of sour taste on swallowing function, Pelletier and Lawless (2003) tested 2.7% citric acid in water (concentration estimated to be equivalent in sourness to the acid level presented in the study of Logeman et al.) on a separate group of neurogenic dysphagic patients using fiberoptic endoscope evaluation study (FEES) instead of VFSS. Citric acid (2.7%) significantly reduced aspiration and penetration compared with water. Furthermore, subjects showed significantly increased spontaneous dry swallows in response to citric acid stimuli. Pelletier and Lawless (2003) suggested that the effect of citric acid on swallowing function could also be due to its trigeminal stimulation (described in detail in Section 2.3).

Further studies on the effect of sour bolus on swallowing were carried out on healthy subjects.

In a very recent study, Wahab et al. (2010) tried to differentiate the olfactory and gustatory stimuli effects of lemon juice on swallowing function. Unfortunately, with their chosen protocol (inhalation through nasal cannula or deposition on the tongue via a filter paper impregnated with lemon juice), the two effects could not be deconvoluted. Nevertheless, their results confirmed that lemon juice could enhance neural swallowing control by enhancement of motor evoked potentials from the submental muscles, and therefore help in triggering swallowing.

Using either intramuscular or sEMG, Ding et al. (2003) and Palmer et al. (2005) reported stronger contraction of muscles when unthickened sour boluses were used compared with water boluses. Palmer et al. (2005) observed that activation of the mylohyoid, anterior belly of the digastrics, and geniohyoid muscles were more closely coordinated during a sour bolus swallow. A sour bolus may stimulate the oral muscles resulting in stronger and quicker contractions during the swallow. The authors argued that “it is likely that the changes in videofluoroscopic events, such as rapid transit times noted in Logemann et al.'s study were, at least in part, the results of quicker and more tightly activated muscle activity from the increased oropharyngeal sensory stimulation.” The stronger muscle contraction in the presence of sour bolus was confirmed by Leow et al. (2007). In the latter case, the authors suggested that the sourness from the citric acid solution activates the nucleus tractus solitarius (NTS), and consequently the nucleus ambiguous motor resulting in greater muscle contraction. Recently, Miura et al. (2009) also demonstrated that unthickened sour solutions increased the power frequency content of submental sEMG. However, Hamdy et al. (2003) found the opposite effects of sour bolus in healthy and stroke populations. Instead of facilitating swallows, liquid cold sour boluses decreased the capacity and speed of swallow. This difference in result may be attributable to their protocol. They performed a water swallow test (WST), in which an individual is asked to drink 50 ml of water from a cup as quickly and as comfortable as possible while being precisely timed. One difference in the WST method used by Hamdy versus other studies is the volume of liquid: 50 ml, instead of much smaller volumes used in the other studies (i.e., 1–3 ml). It is well known that the volume of the bolus can influence swallowing, and that larger volumes are more likely to cause swallowing difficulty (Dantas et al., 1990; Bisch et al., 1994; Butler et al., 2004). Results from Hamdy et al. (2003) may be more relevant for the clinical practices as patients need to drink large volumes to hydrate themselves. Further studies should take into account the true consumption patterns.

One could also advance the hypothesis that the effect of sour bolus could also be explained by its stimulation of salivary glands. Increasing salivary secretion could help in swallowing. Previous work by Mansson and Sandberg (1975) reported a high number of pharyngeal swallows when neutral and sour lozenges were sucked compared in the absence of lozenges. To our knowledge, no other studies have been carried out to quantify the effect of food salivary stimulation on swallowing.

### 2.2. Other Taste Perceptions

There has been significantly less research completed on other taste stimuli in relation to improve swallowing. Furthermore, all the studies were carried out on a healthy population.

Ding et al. (2003) used EMG and showed stronger submental muscle contraction for salty boluses and significantly quicker muscle activation for salty, sweet, and sour unthickened solutions compared with water. Infrahyoid muscle was activated earlier in the sweet and sour taste conditions. The contribution of the shortened start time could be either attributed to an improvement of oral or pharyngeal swallows because submental muscles can be involved both in oral and pharyngeal phases of swallow. The authors concluded that “taste receptors seem to be able to partially affect the sequence of events during swallowing.” Ding et al. (2003) also investigated the combined effect of product consistency [liquid vs semisolid (cottage cheese)] and taste (sweet, salty, and sour) on submental and infrahyoid start time, and showed that the effect of taste was even more prominent. Yahagi et al. (2008) also showed that infusion of 0.3 M NaCl solution on the posterior tongue shortened the swallowing intervals measured by sEMG. Leow et al. (2007) investigated the influence of taste on swallowing apnea, oral preparation time and duration, and amplitude of submental muscle contraction using respiratory airflow and sEMG methods. In contrast to many of the other studies, their study used gelled matrices (5% gelatin). The authors argued that gelled matrices may prolong stimulation of peripheral sensory receptors compared with liquid boluses. No significant influence of taste on the phase location (i.e., during mid expiration, expiration–inspiration, mid inspiration, inspiration–expiration phases) and duration of swallowing apnea was found, in agreement with observations of Butler et al. (2004) and Hiss et al. (2004). However, significant taste effects for oral preparation time, submental sEMG amplitude, and swallow duration were found. Sour and sweet tastants, when tasted alone, exhibited both shorter oral bolus preparation times and shorter duration of submental sEMG contraction compared with bitter and salty tastants. There were no significant differences in oral preparation time and duration of submental sEMG between salty and bitter tastants. The authors suggested that this prolongation in muscle contraction could protect from aspiration. Recently, a Japanese group (Yamada and Uneyama, 2009) reported that umami taste could also facilitate swallowing. However, these papers are brief communications with no direct evidence of the umami effect on swallowing function.

Other research does not support the promotion of swallowing by taste solutions. Chee et al. (2005) reported that liquid solutions of glucose, citrus, salt, and quinine have no effect on swallowing capacity and decrease swallowing speed when compared with water using the WST. However, the authors recognized that activation of sensory receptors could provide significant inputs to the NTS and therefore have an impact on swallowing function. To explain their results, the authors argued that the unfamiliar high intensity of the stimulus may have reduced the amount of solution swallowed. Nevertheless, the concentrations used in this study are not higher than the ones used in previous studies, but once again the bolus volume was large (50 ml). Another study (Kaatzke-McDonald et al., 1996) did not report significant differences among sweet, salted, and distilled water with respect to the latency of evoked swallowing. Salty solutions were slightly more effective than distilled water in the number of swallows evoked. Taste solutions were infused at a rate of 0.1 ml/s for three seconds through a 1.2-mm diameter intraoral tube to minimize any mechanical effects of the stimulus. Swallowing latency and frequency was measured using a laryngograph. The difference in methodology (infusion instead of consumption) may have influenced the result. The authors recognized that their data applied specifically to the faucial pillar area (at the back of the throat) and that this region may be relatively sparsely populated with taste buds.

These conflicting reports on the effect of taste enhancement raise the question whether swallowing could be more driven by the intensity of the taste than by the taste itself. Only Leow et al. (2007) tried to investigate the effect of different tastants with equivalent taste intensities. Pelletier and Dhanaraj (2006) showed that only moderate sucrose, high-salt and high-citric acid concentrations elicited significant higher lingual swallowing pressures compared with the pressures generated by water. These results indicate the potential effect of level of taste intensity.

### 2.3. Olfactory Perception

Retronasal olfactory stimulation using a food like odorant, vanillin, in combination with a congruent sweet liquid solution, has been shown to facilitate swallowing in terms of both the frequency of swallows and the latency of the first swallow (Welge-Lusse et al., 2009). Ultrasound recording of the mouth floor was used to characterize swallowing of a cohort of healthy adults using a single flavor. The authors recognized that further investigations to examine the influence of odor quality, liking, and familiarity on swallowing are needed. Results from Wahab et al. (2010) also seem to indicate that simultaneous stimulation of smell (lemon odor) and taste (lemon taste) could provide an optimal sensory condition to increase swallowing efficiency. Ebihara et al. (2006a) reported on elderly patients with dysphagia that a nasal inhalation of black pepper oil for one minute shortened the latency of the swallowing reflex, and after 30 days also improved the number of swallowing movements for one minute and led to an increase in the substance P, which is known to regulate the reflexible movements of swallowing. Following the work of Ebihara et al. (2006a); Munakata et al. (2008) investigated the effect of inhalation of black pepper oil on pediatric patients with prolonged enteral nutrition. Although complete elimination of the need for enteral nutrition was not achieved, olfactory stimulation with black pepper oil facilitated oral intake in a subset of the young patients (five out of eight). However, no effect on swallowing function was observed using lavender oil. We could hypothesize that the effect of black pepper oil on swallowing function could be more due to its irritant molecules, i.e., piperine than to its odorant molecules. Piperine, a major source of the black pepper oil flavors, is an agonist of the transient receptor potential vanilloid 1 rceptor (TRPV1) involved in trigeminal sensations. Further works are required to understand whether the sensory stimulation is olfactory or trigeminal in nature and to investigate the long-term efficacy of a black pepper oil intervention.

### 2.4. Trigeminal Perceptions

Thermal tactile stimulation therapy, an established technique to treat delayed pharyngeal swallow, confirms the influence of sensory perception on swallowing (Lazzara et al., 1986). Thermal sensitization consists of applying cold (thermal) contact to the base of the anterior faucial arches to prestimulate the oral cavity prior to the introduction of a solution and trigger the swallowing reflex more rapidly. Kaatzke-McDonald et al. (1996) studied the effect of cold (cold vs tepid) and touch (three strokes vs feigned stimulation) stimulation of the anterior faucial pillars on swallowing of healthy subjects. They found that cold touch stimulation evoked a significant increase in swallowing onset time and repetitive frequency of swallowing. The results suggest that there are thermosensitive receptors in the faucial pillars that promote swallowing when stimulated by cold touch. However, at the same time, Ali et al. (1996) reported that normal pharyngeal swallow response is neither facilitated nor inhibited by prior cold tactile stimulation or topical anesthesia of the tonsillar pillars. All these studies were conducted with a probe in a well-defined region of the mouth to prestimulate the swallowing. When the effect of food bolus temperature was investigated, few significant effects of a cold bolus on swallowing function were observed. Bisch et al. (1994) observed that only a group of first-time stroke patients (mild dysphagia) compared with normal subjects and neurologically impaired patients (strong dysphagia) exhibited significantly shorter pharyngeal response time and laryngeal elevation and earlier laryngeal closure with 1-ml cold liquid boluses. Cold stimulation has been much more studied than hot stimulation. Preliminary results from Miyaoka et al. (2006) seem to indicate that thickened water to a creamy consistency (8% Mousse up®, Nisshin Science Clinical Foods, Japan) at 50°C activate less suprahyoid muscles for a healthy population.

Hot and cold perceptions can also be evoked by chemical agents through the stimulation of the trigeminal nerve. Ebihara et al. (2005, 2006b) reported interesting post effects of trigeminal agents on swallowing for institutionalized elderly patients. They showed that the consumption of capsaicin (a hot pepper extract inducing a hot perception) lozenge or of menthol solution (inducing a cold perception) before swallowing stimulation (i.e. injection of 1 ml distilled water into the pharynx through a nasal catheter) significantly improved swallowing reflex. Swallowing was identified by submental EMG activity and visual observation of characteristic laryngeal movement. The latency swallowing response was significantly shortened after 4 weeks consumption of a capsaicin lozenge and in a concentration-dependent manner by the menthol. The reported effect of black pepper oil on the swallowing function also suggests a trigeminal triggering effect on swallowing. It would be interesting to investigate the effect of trigeminal agents such as capsaicin, pepper and menthol incorporated in foods.

Pelletier and Lawless’ (2003) previously discussed work also suggested that trigeminal perception could help in swallowing triggering. High-citric acid concentration is known to elicit chemesthesis.

Another promising trigeminal stimulation to trigger swallowing is the carbonation of drinks. Bulow et al. (2003) showed during a therapeutic VFSS that carbonated liquid, reduced penetration/aspiration and pharyngeal transit time. Pharyngeal retention was also significantly reduced. The authors explained these results in terms of the stimulating effect of molecules from the carbonic acid on faucial isthmus receptors in the mouth, leading to quicker activation of the solitary tract nucleus in the medulla oblongata of the brain stem. Recent results from Miura et al. (2009) confirm these observations with healthy subjects by showing that carbonation significantly increases the spectrum-integrated value of the total power components measured by sEMG.

### 2.5. Texture Perception

One can pose the question as to whether or not the effect of the viscosity on improving swallowing in patients with dysphagia is related to the physical properties of food (better cohesiveness/elasticity) and/or to an enhanced perception. Smith et al. (1997, 2006) evaluated the physical and perceived viscosity of samples with different proportions of water and corn syrup. The relation between physical viscosity and orally perceived viscosity can be explained as a power law: *s* = *kη^β^*, where “*s*” is the psychophysical magnitude, “η” is the viscosity of the physical stimulus, “*k*” is a constant determined by the choice of unit selected for measurements and “*β*” the exponent. Smith et al. (1997) found an exponent for oral perception of fluid viscosity of around 0.3, i.e., the oral sensation of viscosity grows at approximately a third of the rate of the actual viscosity. They suggested a well-controlled viscosity property for thickened liquids may not be necessary, due to the low viscosity sensitivity. However, studies were conducted on Newtonian fluids (water and corn syrup mixed in different proportions), which do not behave like fluids prepared with commercial thickening agents, which most of them are shear thinning fluids. Furthermore, this study was designed to investigate the ability of normal young adults to sensorially identify Newtonian fluid of specified viscosities. Patients with dysphagia may have another perception threshold. To our knowledge, no other results were found to be published on the viscosity perception and its effect on triggering swallowing.

## 3. CONCLUSIONS

The current review highlights the important role played by food sensory properties in promoting swallowing. Most of the literature on taste perception has been focused on the important role played by sour perception. Whereas less research has been completed on the remaining basic tastes. There are conflicting reports that demonstrate both an improvement and no effect across tastants. Further research will be required to conclude on their effects. Few studies have been carried out on the effect of trigeminal, olfactory, or texture perception on swallowing, yet there is enough to suggest that it would be worth a deeper investigation of these effects.

It may be that a combination of approaches is required to promote swallowing. Sciortino et al. (2003), using sEMG, showed that latency to swallow specific activity was significantly shorter following mechanical+cold+gustatory condition compared with no stimulation in a healthy population. The treatment condition that employed all three stimuli together was the only means to produce a significantly quicker average initiation of swallow activity. Furthermore, most of the studies on sensory effects on swallowing were investigated with liquid solutions, which are known to be difficult to swallow. Using thickened solutions may be more relevant to clinical practices.

Some studies have been carried out on healthy and others on patients with dysphagia (mainly neurogenic patients), and the role of sensory stimuli may be quite different in “normal swallowers” compared with patients with dysphagia and within the different dysphagic populations (elderly, neurogenic, strokes patients….). More systematic research is needed on the role of sensory input in normal swallowers (of various ages) and patients with dysphagia (of various etiologies).

These effects do not appear to be linked to food liking. A multidisciplinary approach with appropriate psychophysical methods, patient preference tests (individual liking and cultural differences), and well-controlled swallowing measurements coupled with different methods such as EMG, FEES, accelerometry, and ultrasound (avoiding VFSS due to its impact on taste) as well as food volumes and exposures relevant to clinical practices (i.e., big volumes and number of exposures) has the potential to improve our understanding on swallowing function.

## References

[cit0001] (1996). Influence of cold stimulation on the normal pharyngeal swallow response. Dysphagia..

[cit0003] (2010). Enhancing effects of flavored nutritive stimulation cortical swallowing network activity. Am. J. Physiol. Gastroinstestinal Physiol.

[cit0004] (1994). Pharyngeal effects of bolus volume, viscosity, and temperature in patients with dysphagia resulting from neurologic impairment and in normal subjects. J. Speech Hearing Res.

[cit0005] (2003). Videoradiographic analysis of how carbonated thin liquids and thickened liquids affect the physiology of swallowing in subjects with aspiration on thin liquids. Acta Rheologica..

[cit0006] (2004). Effect of viscosity, taste, and bolus volume on swallowing apnea duration of normal adults. Otolaryngol Head Neck Surg.

[cit0007] (2005). The influence of chemical gustatory stimuli and oral anaesthesia on healthy human pharyngeal swallowing. Chem. Senses.

[cit0008] (1990). Effect of swallowed bolus variables on oral and pharyngeal phases of swallowing. Am. J. Physiol.

[cit0009] (2003). The effects of taste and consistency on swallow physiology in younger and older healthy individuals: A surface electromyographic study. J. Speech Lang. Hear. Res.

[cit0010] (2006a). A randomized trial of olfactory stimulation using black pepper oil in older people with swallowing dysfunction. Am. Geriatr. Soc.

[cit0011] (2006b). Effect of menthol on the triggering of the swallowing reflex in elderly patients with dysphagia. Br. J. Clin. Pharmacol.

[cit0012] (2005). Capsaicin troche for swallowing dysfunction in older people. J. Am. Geriatr. Soc.

[cit0013] (2009). Effect of barium sulphate contrast medium on rheology and sensory texture attributes in a model food. Acta Radiologica.

[cit0014] (2005). Thickened liquids: Practice patterns of speech-language pathologists. Am. J. Speech Lang. Pathol.

[cit0015] (2003). Modulation of human swallowing behaviour by thermal and chemical stimulation in health and after brain injury. Neurogastroenteroly Motil.

[cit0016] (2004). Effects of age, gender, bolus volume, bolus viscosity, and gestation on swallowing apnea onset relative to lingual bolus propulsion onset in normal subjects. J. Speech Lang. Hear. Res.

[cit0017] (1996). The effects of cold, touch, and chemical stimulation of the anterior faucial pillar on human swallowing. Dysphagia..

[cit0018] (1986). Impact of thermal stimulation on the triggering of the swallowing reflex. Dysphagia.

[cit0019] (2007). The influence of taste on swallowing apnea, oral preparation time, and duration and amplitude of submental muscle contraction. Chem. Senses.

[cit0020] (1995). Effects of a sour bolus on oropharyngeal swallowing measures in patients with neurogenic dysphagia. J. Speech Hear. Res.

[cit0021] (2003). Texture and flavors characteristics of beverages containing commercial thickening agents for dysphagia diets. J. Food Sci.

[cit0022] (2001). The effect of compliance on clinical outcomes for patients with dysphagia on videofluoroscopy. Dysphagia..

[cit0023] (2008). Sensory stimulation activates both motor and sensor components of the swallowing system. NeuroImage.

[cit0024] (1975). Salivary stimulus and swallowing in man. Laryngoscope.

[cit0025] (2006). Sensory characteristics of beverages prepared with commercial thickeners used for dysphagia diets. J. Am. Dietetic Assoc.

[cit0026] (2006). Modulation of human cortical swallowing motor pathways after pleasant and aversive taste stimuli. Am. J. Physiol. Gastrointest. Liver Physiol..

[cit0027] (2009). Effects of taste solutions, carbonation and cold stimulus on the power frequency content of swallowing submental surface electromyography. Chem. Senses.

[cit0028] (2006). Influences of thermal and gustatory characteristics on sensory and motor aspects of swallowing. Dysphagia.

[cit0029] (2008). Olfactory stimulation using black pepper oil facilitates oral feeding in pediatric patients receiving long term enteral nutrition. Tohoku J. Exp. Med..

[cit0002] National Dysphagia Diet Task Force (2002). National Dysphagia Diet: Standardization for Optimal Care. Chicago, IL: American Dietetic Association

[cit0030] (2005). Effect of a sour bolus on the intramuscular electromyographic (EMG) activity of muscles in the submental region. Dysphagia.

[cit0031] (1997). A comparison of consistency and taste of five commercial thickeners. Dysphagia.

[cit0032] (2006). The effect of taste and palatability on lingual swallowing pressure. Dysphagia.

[cit0033] (2003). Effect of citric acid and citric-acid-sucrose mixtures on swallowing in neurogenic oropharyngeal dysphagia. Dysphagia.

[cit0034] (2003). Effect of mechanical, cold, gustory, and combined stimulation to the human anterior faucial pillars. Dysphagia.

[cit0035] (1997). Oral sensory discrimination of fluid viscosity. Dysphagia..

[cit0036] (2006). Oral and oropharyngeal perceptions of fluid viscosity across the age span. Dysphagia.

[cit0037] (2010). Sensory input pathways and mechanisms in swallowing: A review. Dysphagia.

[cit0038] (2007). Functional oropharyngeal sensory disruption interferes with the cortical control of swallowing. BMC Neurosci.

[cit0039] (2009). Possible applications of umami taste to improve eating disorders. J. Health Sci.

[cit0040] (2010). Effects of olfactory and gustatory stimuli on neural excitability for swallowing. Physiol. Behavior.

[cit0041] (2009). Swallowing is differentially influenced by retronasal compared with orthonasal stimulation in combination with gustatory stimuli. Chem. Senses.

[cit0042] (2008). Facilitation of voluntary swallowing by chemical stimulation of the posterior tongue and pharyngeal region in humans. Neurosci. Lett.

[cit0043] (2009). Foreword. J. Health Sci.

